# Complementary RNA amplification methods enhance microarray identification of transcripts expressed in the *C. elegans *nervous system

**DOI:** 10.1186/1471-2164-9-84

**Published:** 2008-02-19

**Authors:** Joseph D Watson, Shenglong Wang, Stephen E Von Stetina, W Clay Spencer, Shawn Levy, Phillip J Dexheimer, Nurith Kurn, Joe Don Heath, David M Miller

**Affiliations:** 1 Department of Cell and Developmental Biology, Vanderbilt University, Nashville, TN 37232-8240, USA; 2 Graduate Program in Neuroscience, Center for Molecular Neuroscience, Vanderbilt University, Nashville, TN 37232-8548, USA; 3 NuGEN Technologies, Inc., San Carlos, CA 94070, USA; 4 Department of Biomedical Informatics, Vanderbilt University, Nashville, TN 37232-8240, USA

## Abstract

**Background:**

DNA microarrays provide a powerful method for global analysis of gene expression. The application of this technology to specific cell types and tissues, however, is typically limited by small amounts of available mRNA, thereby necessitating amplification. Here we compare microarray results obtained with two different methods of RNA amplification to profile gene expression in the *C. elegans *larval nervous system.

**Results:**

We used the mRNA-tagging strategy to isolate transcripts specifically from *C. elegans *larval neurons. The WT-Ovation Pico System (WT-Pico) was used to amplify 2 ng of pan-neural RNA to produce labeled cDNA for microarray analysis. These WT-Pico-derived data were compared to microarray results obtained with a labeled aRNA target generated by two rounds of In Vitro Transcription (IVT) of 25 ng of pan-neural RNA. WT-Pico results in a higher fraction of present calls than IVT, a finding consistent with the proposal that DNA-DNA hybridization results in lower mismatch signals than the RNA-DNA heteroduplexes produced by IVT amplification. Microarray data sets from these samples were compared to a reference profile of all larval cells to identify transcripts with elevated expression in neurons. These results were validated by the high proportion of known neuron-expressed genes detected in these profiles and by promoter-GFP constructs for previously uncharacterized genes in these data sets. Together, the IVT and WT-Pico methods identified 2,173 unique neuron-enriched transcripts. Only about half of these transcripts (1,044), however, are detected as enriched by both IVT and WT-Pico amplification.

**Conclusion:**

We show that two different methods of RNA amplification, IVT and WT-Pico, produce valid microarray profiles of gene expression in the *C. elegans *larval nervous system with a low rate of false positives. However, our results also show that each method of RNA amplification detects a unique subset of *bona fide *neural-enriched transcripts and thus a wider array of authentic neural genes are identified by the combination of these data sets than by the microarray profiles obtained with either method of RNA amplification alone. With its relative ease of implementation and greater sensitivity, WT-Pico is the preferred method of amplification for cases in which sample RNA is limiting.

## Background

**Figure 1:**
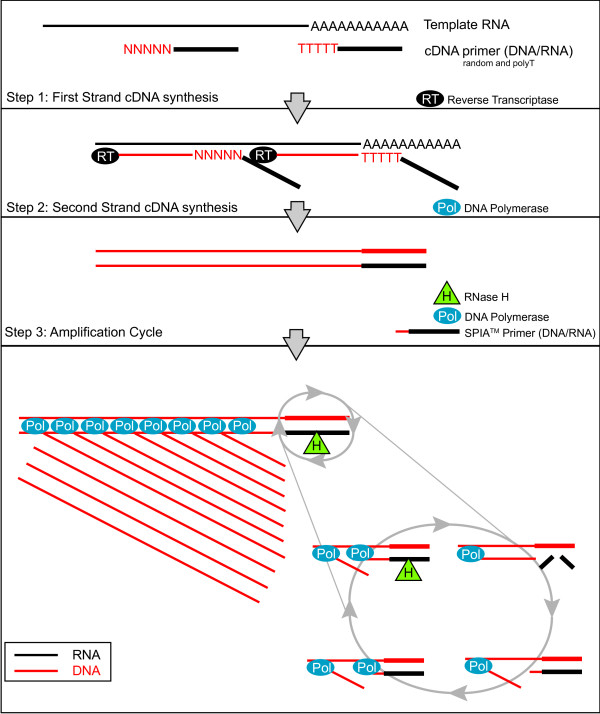
**Diagram of the ribo-SPIA process for the synthesis of sscDNA**. First strand cDNA is generated from template RNA using reverse transcriptase (RT) and two types of chimeric primers, random and oligo(dT), containing an RNA overhang (Step 1). DNA Polymerase is added to the reaction to generate second strand cDNA (Step 2). sscDNA is amplified from the dscDNA template in a cycle in which a SPIA™ primer (DNA/RNA hybrid) anneals to the template, DNA Polymerase begins duplicating the cDNA, the RNA portion of the primer degraded by RNase H (which only degrades RNA when it is in a duplex with DNA), thus allowing another SPIA™ primer to bind to the template and restart the reaction (Step 3).

The human brain is comprised of diverse classes of neurons, and many of these neural classes are conserved throughout evolution. Our understanding of the molecular basis for these differences would be greatly advanced by a gene expression map of the nervous system. In principle, this information could be compiled from high density microarray experiments that catalog transcripts expressed in each class of neuron [1,2]. This approach necessarily requires, however, methods for extracting transcripts from individual cell types. Several approaches are now available for overcoming this technical hurdle. Laser Capture Microdissection (LCM) [3] and FACS (Fluorescence Activated Cell Sorting) [4] have been used to isolate specific neurons for RNA extraction. For example, specific GFP-labeled neurons and muscle cells have been obtained by FACS from the nematode, *C. elegans*, for microarray gene expression profiling experiments [5-9]. In several instances, disruption of specific genes included in these data sets revealed key functional roles in the profiled cell type [5]. A recently developed alternative biochemical strategy is also available for extracting RNA from specific cells that may not be readily dissociated for FACS. In this "mRNA-tagging" approach, an epitope-labeled mRNA binding protein (FLAG-PAB-1) is transgenically expressed with a cell-specific promoter. Bound mRNA is then obtained by co-immunoprecipitation with the FLAG-PAB-1 protein [10]. This method has been utilized to profile tissues and cell types in *C. elegans *and in *Drosophila *[8,10-14]. Thus, robust physical and biochemical methods are now available for obtaining mRNA from specific types of neurons in several different organisms. The limited amount of RNA (< 1 ug) available from these approaches, however, typically requires amplification before microarray analysis [15]. One method of amplification, PCR based exponential amplification, can generate microgram quantities of cDNA from as little as 1 ng of total RNA. PCR based methods, however, have been shown to be less reproducible than linear amplification methods, such as In Vitro Transcription (IVT) [16]. IVT has been widely utilized for RNA amplification [17]. In this approach, cDNA is initially synthesized to provide a template for amplification by T7 RNA polymerase. In most cases, two rounds of cDNA synthesis and IVT are required to generate sufficient aRNA (> 10 ug) for microarray hybridization. We have used the IVT method to produce robust gene expression profiles of *C. elegans *neurons and muscle cells [6,8,9,12,18]. In some instances, it was difficult to obtain enough RNA for reliable IVT amplification; in other cases IVT failed for unknown reasons (JDW, SEV and DMM, unpublished data). Thus, we needed a more sensitive and reliable method of RNA amplification. Here we describe microarray results obtained with RNA amplified by WT-Ovation Pico, an isothermal linear amplification system (WT-Pico) [19-22]. For the first step in this protocol (Fig. 1), cDNA is synthesized with a combination of a Poly-dT and random primers. cDNA synthesis primers and the amplification primer are chimeric DNA/RNA oligonucleotides, comprising 3'-DNA and a 5'-RNA sequences. Second strand cDNA synthesis produces a complementary DNA/RNA duplex adjacent to the 1^st ^strand-priming site. Treatment with RNase H selectively removes the RNA sequence from the heteroduplex and provides a unique priming site for cDNA amplification. A chimeric amplification primer hybridizes to this priming site and is extended by DNA polymerase with strand displacement activity to generate a new cDNA strand. The RNA portion of the hybridized primer is removed by RNase H and the cycle is re-initiated by annealing of the amplification chimeric primer (SPIA primer). The net result is synthesis of multiple copies of cDNA in a single amplification step. The WT-Pico system offers the advantage of requiring less time and fewer steps than the IVT amplification [19]. In addition, the DNA target generated by WT-Pico amplification is reported to result in more efficient and specific hybridization with DNA probe sets than the labeled aRNA target produced with IVT amplification [23,24]. We utilized the WT-Pico method to amplify RNA extracted from the *C. elegans *nervous system by the mRNA-tagging method and compared microarray data from this sample to a previously reported pan-neural profile generated with IVT amplification [8]. In our hands, robust microarray data were obtained with WT-Pico amplification from 10-fold less starting RNA than with IVT. In addition, we have confirmed that the WT-Pico cDNA target results in a greater dynamic range and improved signal/noise with more present calls than with the IVT-generated aRNA target. Bioinformatic analysis and *in vivo *expression data from promoter-GFP fusion genes established that the gene expression profile generated with WT-Pico is highly enriched for neuronal transcripts. The microarray data from the pan-neural-derived samples amplified by the IVT and WT-Pico methods identifies 2,173 transcripts with elevated expression in the *C. elegans *nervous system. mRNAs included in this neural-enriched sample encode proteins with a broad array of predicted functions in the *C. elegans *nervous system. Only ~50% of these transcripts, however, are detected as enriched by both methods of RNA amplification. On the basis of this result, we suggest that the IVT and WT-Pico amplification methods show significant nucleotide sequence bias and therefore that, where possible, comprehensive gene expression profiles should be based on more than one method of RNA amplification.

## Results and discussion

### A comparison of two amplification methods, WT-Pico and IVT

**Figure 2:**
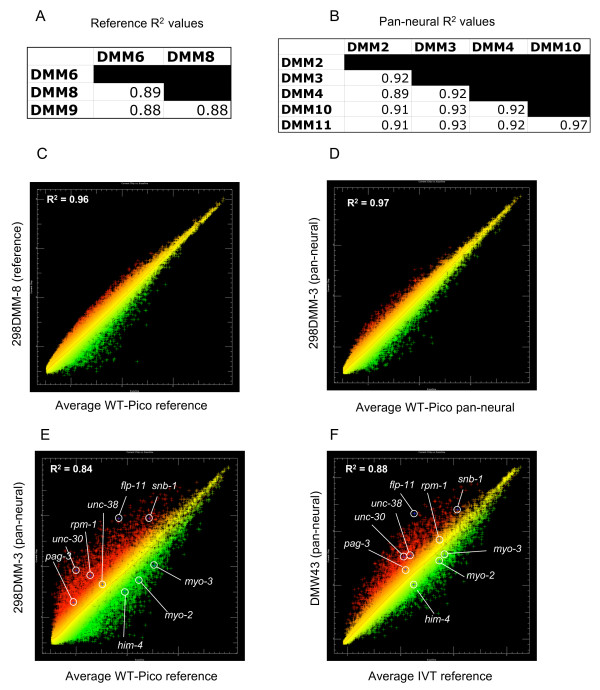
**Microarray data derived from WT-Pico amplified pan-neural samples are reproducible and enriched for neural genes**. **A-B**. Pairwise comparisons of individual hybridizations. Coefficient of determination (R^2^) values for **A**., all pairwise combinations of reference hybridizations and for **B**., all pairwise combinations of larval pan-neural data sets are indicative of reproducible results for both reference and experimental samples. **C**. Scatter plot of intensity values (log_2_) for a representative hybridization (298-DMM-8) (red) isolated from all larval cells (reference) compared to the average intensity of the reference data set (green). **D**. Scatter plot (log_2_) of representative pan-neural hybridization (298-DMM-3) (red) compared to the average intensities for all five pan-neural hybridizations (green). **E**. A representative WT-Pico-amplified pan-neural sample (298-DMM-3) (red) compared to the average of WT-Pico amplified reference samples (green). **F**. A representative IVT-amplified pan-neural sample (DMW43) (red) compared to the average of IVT-amplified reference samples (green). R^2 ^= Coefficient of determination. Selected genes enriched in both the WT-Pico-amplified and IVT-amplified pan-neural samples are: *snb-1*(synaptobrevin), *flp-11 *(neuropeptide); *unc-30 *(homeodomain trancription factor). Transcripts exclusively enriched in the WT-Pico sample include *rpm-1 *(E3 ubiquitin ligase) and *pag-3 *(Zn-finger transcription factor). The nicotinic acetylcholine receptor *unc-38 *is selectively enriched in the IVT data set. Examples of transcripts that are depleted in both pan-neural samples are: *him-4 *(hemicentin, body muscle), *myo-2 *(myosin heavy chain, pharyngeal muscle), *myo-3 *(myosin heavy chain, body wall muscle).

We used the WT-Pico method (Fig. 1) [20] to amplify RNA obtained from all *C. elegans *neurons ("pan-neural") by the mRNA-tagging method [8,10]. Microarray data were generated from five independent pan-neural RNA samples. A companion reference data set was obtained with three replicates of RNA from all *C. elegans *cells. These results were compared to microarray data previously obtained from IVT-amplified samples [8]. Eight pan-neural and reference data sets were produced for each amplification method (Table 1). All WT-Pico amplification reactions were performed with 2 ng of starting RNA whereas the IVT amplifications utilized 25 ng of sample RNA. Comparisons of signal intensities generated from independent replicates showed that the WT-Pico-amplified pan-neural and reference samples are reproducible. For example, the coefficient of determination for the five pan-neural WT-Pico amplified samples, R^2 ^= 0.96, compares favorably to an R^2 ^= 0.98 for the three IVT-generated pan-neural profiles (Fig. 2a–d). Thus, these R^2 ^values are indicative of highly reproducible data sets.

**Table 1:** Hybridization and amplification summaries for WT-Pico and IVT amplifications

**Amplification method and sample type**	**Starting Material (ng)**	**Average Yield (ug)**	**Average yield/ng**	**No. of Chips**	**Scale Factor**	**Average Intensity Values**	**Affymetrix average present calls/chip**
IVT neural	25	44.8	1.8	3	4.4	817.0	41.4
IVT reference	25	38.8	1.6	5	5.4	898.6	42.1
WT-Pico neural	2	10.5	5.3	5	9.6	841.2	**56.1**
WT-Pico reference	2	7.6	3.8	3	16.4	991.2	46.7

We measured other parameters derived from the microarray data to compare the performance of the WT-Pico vs IVT-amplified targets. The *C. elegans *Affymetrix Gene Chip includes 22,499 probe sets. On the Affymetrix Gene Chip, each Perfect Match (PM) oligonucleotide is paired with a MisMatch (MM) probe that includes a single base pair substitution. The hybridization intensity of each MM probe is subtracted from that of the paired PM probe to correct for stray signal. An overall PM vs MM discrimination score for the probe set is calculated from these values to distinguish between present, Marginal or Absent transcripts [25] (See methods). Overall, hybridizations with the WT-Pico amplified sample resulted in a greater number of present calls than with the IVT target (Table 1). For example, an average of 56% (~12,600) of probe sets were scored as present in the pan-neural profiles obtained by WT-Pico amplification whereas an average of only 41% (~9,200) of probe sets were called present in the IVT-generated pan-neural data set (Table 1) (p < 0.05) (See Methods). A similar difference is noted from a comparison of the fraction of transcripts detected on all of the pan-neural arrays. In this case, WT-Pico amplification identifies 9,198 present transcripts whereas the IVT-derived target reports 7,382 present calls (Table 2) [See Additional file 1]. The removal from these comparisons of duplicate probe sets (i.e. probe sets for the same gene) resulted in a final difference of 7,409 present genes in the WT-Pico-derived data set vs 6,354 present calls in the IVT profile (Table 2).

**Table 2:** Total number of transcripts identified by RNA amplification

**Amplification method and sample type**	**Total number of present probesets**	**Total number of present genes**
IVT neural	7382	6354
IVT reference	7325	6302
WT-Pico neural	**9198**	**7409**
WT-Pico reference	7771	6238

**Figure 3:**
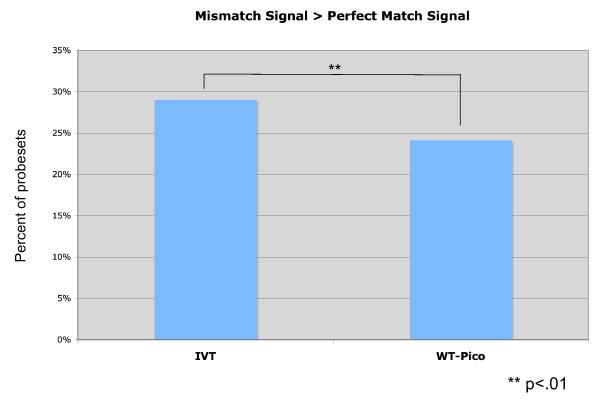
**Signal/Noise is enhanced with WT-Pico vs IVT-amplified targets**. MisMatch (MM) signals were higher than Perfect Match (PM) intensities more frequently with IVT-amplified samples than with WT-Pico-amplified targets. Chart represents average MM/PM signal intensities from all microarray samples.

The greater number of present calls derived from the WT-Pico data sets is correlated with the finding that the WT-Pico target results in relatively less mismatch hybridization than the IVT sample [23]. For the combined IVT-amplified pan-neural and reference samples, we find that 29 ± 0.5% of MM signals exceed the paired PM value whereas only 24 ± 1% of the WT-Pico derived signals show MM > PM ratios (p < 0.01) (Fig. 3). Similar results have been noted previously and attributed to the finding that mismatched RNA:DNA heteroduplexes are thermodynamically more stable than comparable DNA:DNA hybrids [23,24].

### Neuron-enriched transcripts are identified in both the WT-Pico and IVT-amplified samples

To test the ability of the WT-Pico-amplified sample to detect differentially expressed transcripts, the pan-neural data set was compared to the reference profile obtained from all cells (see Methods). As expected, scatter plots reveal significant differences between these data sets with 1,625 transcripts showing elevated intensity values in the pan-neural sample vs 1,325 depleted mRNAs (Fig. 2e) [See Additional file 2]. (Similar results (Fig. 2f) were obtained by the IVT amplification method [8]). As an independent test of the validity of these data, the list of 1,625 transcripts showing elevated intensity values in the WT-Pico derived Pan-neural data set (i.e., "enriched genes") was compared to WormBase to identify the subset of transcripts previously described as expressed in neurons [8].

**Figure 4:**
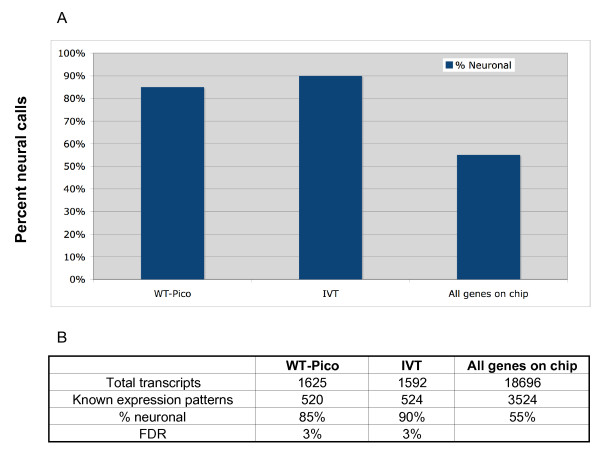
**Established neuron-expressed transcripts are highly represented in WT-Pico and IVT-amplified pan-neural samples**. **A**. Histrogram showing percent annotated genes in microarray data sets with known *in vivo *expression in neurons. All genes with known cellular expression patterns listed in WormBase were used for this comparison. Note significant enrichment for neuronal genes in both the WT-Pico and IVT-amplified pan-neural samples (85–90%) relative to the fraction of annotated genes in WormBase (55%) that show some expression in the *C. elegans *nervous system. **B**. Summary of expression data from transcripts enriched in WT-Pico and IVT-amplified pan-neural samples. (FDR = False Discovery Rate, see Methods).

This analysis revealed 520 transcripts in the WT-Pico-amplified data set with known expression patterns *in vivo*. Of these, 85% are annotated in WormBase as expressed in neurons (Fig. 4). This finding is comparable to the observation that 90% of the 524 transcripts in the enriched IVT-amplified pan-neural profile with expression data in WormBase are also detected in neurons [See Additional file 3]. In both cases, the microarray profiles show a significant bias for authentic neuronal transcripts as only 55% of all genes with expression patterns listed in WormBase are neuronal (Fig. 4) [See Additional file 4]. These findings confirm that both the WT-Pico and IVT amplification methods detect transcripts that are differentially expressed in the *C. elegans *nervous system.

**Figure 5:**
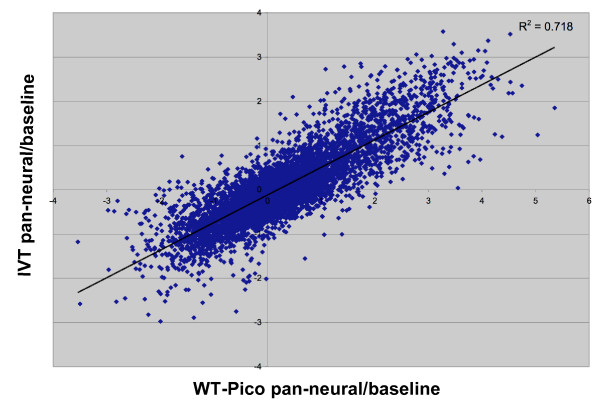
**Correlation of differential gene expression between WT-Pico and IVT-amplified samples**. Average ratios (log_2 _of pan-neural/reference) of RMA-normalized intensity values are plotted for transcripts scored as present in all eight pan-neural arrays (WT-Pico = 5, IVT = 3). A linear least squares fit (black line) and coefficient of determination (R^2^) are shown.

To estimate the concordance of these data, we compared normalized intensity values for differentially expressed transcripts identified by each method. Log_2 _of the IVT pan-neural/reference ratio was plotted versus that of the WT-Pico/reference (Fig. 5) for probe sets with present calls in all of the pan-neural samples (see Methods) [See Additional file 5]. The R^2 ^value of 0.72 is indicative of significant correlation between these two amplification methods for the subset of transcripts that are detected in both, an outcome similar to that seen in previous comparisons of WT-Pico vs IVT [20,24].

**Figure 6:**
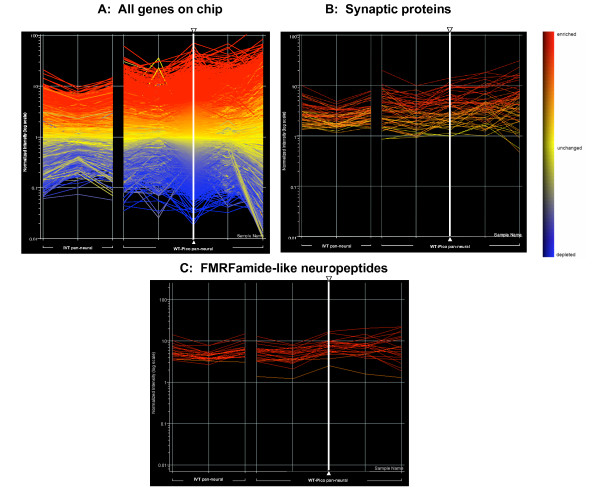
**Similar overall patterns of differential gene expression are observed for WT-Pico and IVT-amplified pan-neural samples**. A. Line graphs display log_10 _of relative intensity values (pan-neural/average reference) for all probes sets on the Affymetrix array. Each vertical line corresponds to a single pan-neural replicate (IVT = 3, WT-Pico = 5). Colors from the heat map at right are defined by the WT-Pico sample denoted by the bold vertical white line with arrowheads (298-DMM4). **B**. Line graph showing that most of the 53 [8] synaptic vesicle-associated genes are enriched (red) in the WT-Pico derived samples. **C**. 22 of 23 FMRFamide-like neuropeptides (flps) enriched in the IVT sample also show elevated relative intensity values (red) in the WT-Pico data set.

We expanded this comparison to consider all probe sets on the *C. elegans *chip. These results are depicted in Fig. 6 in the form of a line graph in which the intensity values for all three of the IVT pan-neural replicates and for the five WT-Pico pan-neural samples are normalized against the corresponding average reference intensities. Lines are color coded as enriched (red), depleted (blue) or unchanged (yellow) relative to the reference. Colors for each gene are fixed by the relative values of sample #3 (vertical bold white line) in the WT-Pico data set. This global analysis suggests an overall trend in which transcripts detected by both methods show similar patterns of differential expression. For example, 53 transcripts enriched in the IVT-derived pan-neural sample encode proteins with established or likely functions in neurotransmitter release at the synapse [8]. 37 (70%) of these genes are also enriched in the WT-Pico pan-neural data set and essentially all of these transcripts show intensity values greater than or equal to the reference (Fig. 6B). Similar results were obtained for transcripts encoding FMRFamide-like proteins (flps), a large family of peptide neurotransmitters that are largely restricted to the *C. elegans *nervous system (Fig. 6C) [26]. In addition to identifying similar trends in the relative intensity values of specific transcripts obtained by both methods, these line graphs also reveal a difference in the apparent overall spread of hybridization signals with the WT-Pico results showing a significantly larger dynamic range of differential expression vs the IVT data set (Fig. 6A, B). A similar result was obtained in a previous comparison of IVT vs WT-Pico derived microarray data [23].

### WT-Pico and IVT amplified targets reveal distinct neural transcripts

**Figure 7:**
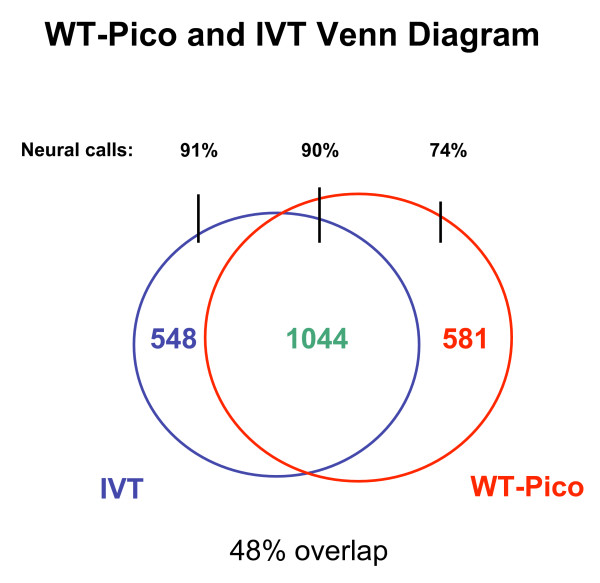
**Comparison of IVT and WT-Pico derived data sets reveals differentially enriched transcripts**. A Venn diagram denoting 1044 transcripts enriched in both data sets, 548 genes selectively elevated in the IVT-derived profile, and 581 genes exclusively enriched in the WT-Pico-amplified sample. The percentage of neuron-expressed genes in each group with annotated expression in WormBase is noted as "Neural calls."

**Figure 8:**
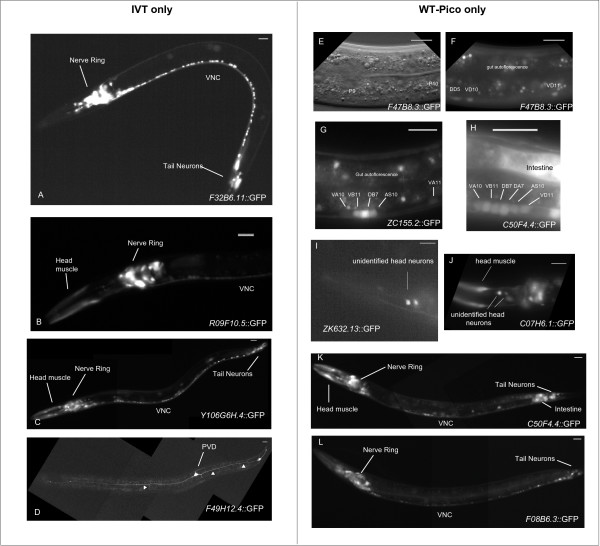
**Promoter-GFP reporter genes confirm neural expression of transcripts from pan-neural enriched data sets**. Transgenic animals expressing GFP reporters for representative transcripts exclusively enriched in either the IVT derived data set (**A-D**) or the WT-Pico-amplified sample (**E-L**). **A**. *F32B6.11*::GFP is expressed throughout the *C. elegans *nervous system including neurons associated with the Nerve ring in the head, motor neurons throughout the Ventral Nerve Cord (VNC) and in tail ganglia. **D**. *F49H12.4*::GFP is selectively expressed in PVD nociceptive neuron and in two additional neurons in the tail region. Note the highly branched PVD dendritic architecture (arrowheads). **E**. Differential Interference Contrast (DIC) image of midbody region of 2^nd ^stage larva (**F.) **expressing *F47B8.3*::GFP in GABAergic motor neurons (DD5, VD10, VD11) in the ventral nerve cord. P9 and P10 denote landmark hypodermal blast cells. **G-H**. *ZC155.2*::GFP and *C50F4.4*::GFP are expressed in VNC motor neurons (e.g. VA10, VB11, etc). Anterior to left, Ventral down. VNC (Ventral Nerve Cord).

A comparison of the pan-neural enriched transcripts detected in these microarray experiments identifies a core group of 1,044 genes that are detected by both the IVT and WT-Pico methods (Fig. 7) [See Additional file 6]. This analysis also revealed, however, that a comparable number of transcripts is selectively enriched in either the WT-Pico or IVT derived data sets; 581 transcripts are detected as enriched in the WT-Pico pan-neural sample but not in the IVT data set whereas 548 genes are specifically enriched in the IVT pan-neural profile but not in the WT-Pico pan-neural data set [See Additional file 6]. These findings were validated by comparison to independently derived data that measures the expression and function of these genes in the *C. elegans *nervous system *in vivo*. First, we established that a majority of genes in either the IVT-only or WT-Pico-only pan-neural enriched data sets with known expression patterns in WormBase are annotated as expressed in neurons (Fig. 7). Additional genetic data have established specific neural functions for a subset of these differentially detected genes. For example, the WT-Pico-only subset of pan-neural enriched transcripts includes *rpm-1*, an E3 ubiquitin ligase that regulates synaptic assembly (Fig. 2e) [27,28]. Similarly, the transcripts encoding the transmembrane protein MIG-13, which affects migration of the Q neuroblast and its descendants, is enriched exclusively in the IVT data set (Fig. 2f) [29]. These findings suggest that each method of RNA amplification may result in the detection of a unique subset of *bona fide *pan-neural enriched genes. We tested this idea by constructing GFP reporters for a representative set of genes listed in either the WT-Pico only or IVT pan-neural only enriched data sets (Table 3). In this approach, the upstream promoter or regulatory region of a specific gene is fused to GFP and reintroduced into the organism to monitor expression in the intact animal (see Methods). Nine transgenic lines were constructed from the WT-Pico-only data set. Neuronal GFP expression was confirmed in all 9 of these lines with reporters for two genes (*ZC155.2, C07H6.1*) showing GFP expression exclusively in neurons (Fig. 8). Similarly, 7 out of 8 (Table 3) GFP reporters for genes in the IVT-only neural enriched data set show expression in *C. elegans *neurons *in vivo *(Fig. 8). Thus, these results support the conclusion that the pan-neural enriched data sets generated by each of these methods are reliably detecting transcripts expressed in the *C. elegans *nervous system.

**Table 3:** Expression of promoter-GFP reporters for transcripts enriched in larval pan-neural or A-class motor neuron data sets.

**Cosmid**	**Gene**	**KOG (other description)**	**IVT Fold Change**	**WT-Pico Fold Change**	**In Neurons?**
*IVT only*					
F30F8.2		Glutaminase	1.6	--	√
F32B6.11		Unnamed Protein	2.5	--	√
F49H12.4		(novel)	2.6	--	√
H01A20.1	*nhr-3*	Nuclear Hormone Receptor	1.7	--	√
R09F10.5		(novel)	2.3	--	√
T19C4.5		(novel)	2.0	--	No expression
W01H2.3	*rab-37*	(Rab GTPase)	6.9	--	√
Y106G6H.4		Unnamed Protein	1.9	--	√
*WT-Pico only*					
C50F4.4		(novel)	--	2.0	√
F08B6.3		Reticulocalbin, calumenin, DNA supercoiling factor, and related Ca2+-binding proteins of the CREC family (EF-Hand protein superfamily)	--	2.7	√
K10B2.4		Predicted membrane protein	--	1.6	√
ZK632.13	*lin-52*	Uncharacterized conserved protein	--	2.9	√
C07H6.1	*lig-4*	ATP-dependent DNA ligase IV	--	2.9	√
F08G12.1		GTPase Rab1/YPT1, small G protein superfamily, and related GTP-binding proteins	--	3.6	√
F47B8.3		Glutaredoxin-related protein	--	2.0	√
T20G5.10		General control of a.a. synthesis 5-like 1	--	2.7	√
ZC155.2		(putative nucleosome assembly factor)	--	2.8	√

GFP expression was typically determined in L2 larvae. Full expression pattens can be found in Additional file 9.

### The WT-Pico and IVT amplified samples identify *C. elegans *genes with homologs expressed in the mammalian brain

Microarray analysis of the IVT-amplified pan-neural sample detected 1,592 transcripts with elevated expression in *C. elegans *neurons [8] [See Additional file 3]. The independent microarray profile of these samples generated with the WT-Pico method has now identified an additional set of 581 neuron-enriched genes to yield a total of 2,173 transcripts that are highly expressed in the *C. elegans *nervous system (Fig. 7). Thus, the use of two alternative methods of RNA amplification has significantly expanded (~36%) the list of transcripts that are differentially expressed in *C. elegans *neurons. To assess the potential value of these additional data for studies of gene function in the nervous system, we identified a subset of genes in the WT-Pico-only list that are evolutionarily conserved but for which biochemical functions have not been previously assigned. This analysis yielded a total of 39 uncharacterized, highly conserved genes [See Additional file 7]. To determine if these transcripts are also expressed in mammalian neurons, we searched the Allen Brain Atlas, an online *in situ *hybridization database, for evidence of expression in the mouse brain [30]. *in situ *data are available for 27 apparent homologs of the *C. elegans *genes on our list of WT-Pico-only enriched transcripts; 74% of these genes (20/27) show expression in the mouse brain. In the case of the IVT-only enriched transcripts, all seven of the uncharacterized, conserved genes for which *in situ *data are available in the Allen Brain Atlas are annotated as expressed in the mouse brain [8]. These results support the idea that genes that are uniquely detected by one of these amplification methods are likely to encode authentic neural transcripts and that these combined data can provide potentially valuable clues to gene expression in the human brain.

### 3' bias does not account for differentially enriched targets identified by either WT-Pico or IVT

WT-Pico uses a combination of Poly-dT and random priming to amplify RNA. In contrast, the first round of the IVT is limited to Poly-dT priming. We speculated that this inherent difference in the amplification procedures might bias IVT towards probesets near the 3' end of a transcript. To test this hypothesis, each probeset identified as enriched by only IVT or only WT-Pico was mapped with the BLAT tool [31] to a unique chromosomal location in the WS170 assembly. From this position, we calculated the distance from the 3' end of the probeset to the 3' end of the gene it targets [See Additional file 8]. No statistically significant difference was found between the locations of the probesets unique to the WT-Pico method and those unique to the IVT method (p = 0.75). We therefore conclude that differential hybridization of WT-Pico vs IVT-generated targets is not due to a systematic bias of either amplified sample for probe sets near the 3' end of targeted transcripts. It should be noted however, that the probe sets in the GeneChip expression arrays used in this study are largely directed towards the 3'-end of the transcripts and therefore would not detect WT-Pico derived targets originating from more 5' regions. In the future, it will be interesting to examine transcripts that are independently detected with either IVT or WT-Pico-derived samples for potential nucleotide sequences that could exert differential effects on either RNA amplification or target hybridization.

## Conclusion

We have confirmed that the WT-Pico method affords rapid and efficient RNA amplification with a higher fraction of present calls after microarray hybridization than targets amplified by the IVT protocol. The WT-Pico method is also technically easier to implement than IVT and requires significantly less time to perform. Although both approaches generate robust microarray profiles of gene expression in the *C. elegans *nervous system, a significant fraction of authentic neuron-enriched transcripts are uniquely identified by each of these methods of RNA amplification. Thus, the combined result obtained with both amplification strategies provides a more complete picture of neural gene expression than either sample alone. For cases in which RNA is limiting, as in the effort to profile single neuron types from *C. elegans*, the enhanced sensitivity of the WT-Pico method is advantageous.

## Methods

### Nematode strains

Nematodes were grown as described [32]. Strains used to isolate transcripts via mRNA-tagging were N2 (wildtype Bristol strain) and SD1241 (*gaIs153*, *F25B3.3*::FLAG::PAB-1) [8].

### Generating transgenic lines expressing GFP reporter genes

Promoter-GFP fusion genes were obtained from the Promoterome project and transgenic lines generated by microparticle bombardment as described [6]. Additional file 9 contains a list of strains described in this paper.

### mRNA-tagging and RNA amplification

The "in vitro transcribed" or "IVT" microarray data sets used in this paper are described in a previous publication. To generate these data sets, 25 ng of RNA from three pan-neural replicates and from five independent N2 (reference) samples was amplified by the IVT method [8]. 2 ng of these RNAs was amplified using version 1 of the WT-Ovation Pico System, which combines **WT-Ovation™ Pico RNA Amplification System **and target preparation according to fragmentation and labeling section of **Ovation™ Biotin RNA Amplification and Labeling System **as described in the User Guides [33]. Two of the previously prepared reference RNA preparations did not amplify by WT-Pico. Two additional samples were isolated by the mRNA-tagging method from the pan-neural transgenic line, SD1241[8] for WT-Pico amplification to yield a total of five pan-neural replicates and three reference samples for the "WT-Pico" profiles. Thus, six of the eight pan-neural and reference data sets generated by each of the RNA amplification methods (IVT or WT-Pico) were obtained from identical RNA samples. A quantitative comparison of microarray results obtained from the two new pan-neural RNA samples (DMM10 and DMM11) used for the WT-Pico amplification vs the originally isolated pan-neural preparations (DMM2, DMM3, DMM4) also used for the IVT amplification [8] showed a broadly similar distribution of intensity values (R^2 ^> 0.91) (see Fig 2).

### Microarray data analysis

Microarray data were processed as described [8,9]. Briefly, intensity values from each hybridization were scaled vs a global average signal from the same array and normalized by Robust Multichip Average analysis (RMA) [34]. To identify differentially expressed transcripts, normalized intensity values from the pan-neural data sets were compared to a reference (from all larval cells) using Significance Analysis of Microarray software (SAM) [35]. A two-class unpaired analysis of the data was performed to identify neuron-enriched genes. Pan-neural enriched transcripts in the IVT and WT-Pico-derived data set were defined as 1.5X elevated vs the reference at a False Discovery Rate (FDR) = 3%. An earlier report describing the IVT-amplified pan-neural data set utilized a more stringent FDR of 1% and therefore identified a smaller number of pan-neural enriched transcripts (1,562 vs 1,592 in this study) ([8], this work). The data discussed in this manuscript are available in the NCBI Gene Expression Omnibus, series accession number GSE9485.

### Annotation of data sets and additional data analysis

Annotation was performed as previously described using WormBase Release 170 . Affymetrix GeneChip Operating Software (GCOS) was used to calculate the average number of present calls for each probe set (Table 1) [25]. Present calls listed in Table 2 and used to calculate Fig. 5 were identified with a Perl script (consensus.pl) [See Additional file 10]. For a given sample (e.g. IVT pan-neural) a transcript was scored "present" if called present in all replicates. For Fig. 5., RMA-normalized intensities for these present genes were averaged across all replicates. Average pan-neural/reference intensities were calculated for WT-Pico and IVT data and log_2 _transformed. The coefficient of determination (R^2^) for the resulting scatter plot was calculated in Microsoft Excel.

Mismatch (MM) intensities were compared against Perfect Match (PM) intensities for both pan-neural and reference samples using the Bioconductor [36] Affy package [36] for Fig. 3.

RMA normalized intensity values for all data sets were imported into GeneSpring GX 7.3 [37] to generate the line graphs shown in Fig. 6. Each Experimental data set was normalized vs the average intensity value for each probe set in the corresponding reference data set and plotted as log (Experimental/reference). Each vertical line represents an individual replicate for each Experimental sample.

p-values for total yield, number of present genes, and perfect vs mismatch probes were calculated using a two-tailed t-test with unequal variance.

### 3' bias analysis

IVT-only and WT-Pico only enriched transcripts were examined for 3' bias. C_elegans_target.fa was downloaded from Affymetrix. This file contains the reference sequence for each probeset on the array. The file Caenorhabditis_elegans.WB170.45.dna.seqlevel.fa was downloaded from Ensembl (Ensembl 45, based on Wormbase 170). Probesets were aligned to chromosomes using BLAT [31]. Where multiple alignments were found, the alignment that covered the longest portion of the probeset sequence was chosen. The genes and chromosomal locations of those genes were downloaded using Ensembl 45. The probeset distance from the 3' end of the gene was calculated. For genes on the (+) strand, the distance is given as (Gene End) – (Probeset End). For genes on the (-) strand, the distance is given as (Probeset Start) – (Gene Start). For probesets that correspond to multiple genes, the gene with the smallest absolute value of 3' distance was chosen. p-value was calculated using a 2-tailed t-test with equal variance.

### Microscopy and identification of GFP expressing cells

GFP-expressing animals were visualized by Differential Interference Contrast (DIC) and epifluorescence optics in either a Zeiss Axioplan or Axiovert compound microscope. Digital images were recorded with CCD cameras (ORCA I or ORCA ER, Hamamatsu Corporation, Bridgewater, NJ).

## Authors' contributions

JDW directed the microarray data analysis, generated GFP reporter lines, scored GFP reporters, compiled Worm Base 170 expression data and helped draft the manuscript. SW performed all WT-Pico amplifications. NK and JDH helped design experimental methods and evaluated results. SEV generated GFP reporter lines, scored GFP expression, and helped draft the manuscript. WCS generated additional GFP transgenic lines and helped with data analysis. SL provided advice on experimental strategies and PD performed the analysis of potential differences in the 3' bias of IVT vs WT-Pico amplified samples. DMM oversaw all aspects of the project and drafted the manuscript.

## Supplementary Material

Additional File 1 Genes detected in neurons by WT-Pico and IVT. A list of genes present in 3 of 3 IVT pan-neural samples and 5 of 5 WT-Pico pan-neural samples. Worksheets include genes found multiple times in a single data set (with dups) and a list in which these duplications have been removed (without dups). Click here for file

Additional File 2 WT-Pico pan-neural enriched and depleted genes. These worksheets list the pan-neural enriched genes from WT-Pico and also genes depleted in the WT-Pico data set relative to reference samples. Click here for file

Additional File 3 IVT pan-neural enriched genes. These worksheets contain pan-neural enriched data sets previously published in our lab [8]. This data has been updated from WS140 to WS170 to reflect changes in *C. elegans *annotation. Click here for file

Additional File 4 WormBase 170 (WS170) annotated Affymetrix data. This file contains descriptions of all genes on the Affymetrix *C. elegans* expression array. Click here for file

Additional File 5 Fold change of transcripts present in both WT-Pico and IVT pan-neural data sets. This file identifies all transcripts present in both IVT and WT-Pico pan-neural data sets and also lists the fold change of these transcripts. This list was used to generate the scatter plot from fig. 5. Click here for file

Additional File 6 A comparison of transcripts enriched in IVT and WT-Pico pan-neural data sets. The three worksheets contained in this file list genes found in the IVT-only data set, the WT-Pico-only data set, or genes enriched in both the IVT and WT-Pico data sets. Click here for file

Additional File 7 Uncharacterized conserved genes found in the WT-Pico-only data set. This file contains a list of WT-Pico-only conserved, uncharacterized genes that have available mouse *in situ *data. Click here for file

Additional File 8 3' bias analysis of IVT and WT-Pico data. This file contains raw data and charts used to examine 3' bias in IVT and WT-Pico amplifications. Click here for file

Additional File 9 Full list of GFP reporter lines generated for figure 8. List strain names and full expression patterns of GFP reporter lines built for this analysis. Click here for file

Additional File 10 Present calls perl script. Perl script file used to determine which genes on the Affymetrix array were present in 3 of 3 IVT samples. Click here for file
